# Use of Interventional Radiology in Critically Injured Children Admitted in a Pediatric Intensive Care Unit of a Developing Country

**DOI:** 10.7759/cureus.3922

**Published:** 2019-01-19

**Authors:** Qalab Abbas, Muhammad Tariq Jamil, Anwar Haque, Raza Sayani

**Affiliations:** 1 Pediatrics, Aga Khan University Hospital, Karachi, PAK; 2 Pediatrics, The Indus Hospital, Karachi, PAK; 3 Radiology, Aga Khan University Hospital, Karachi, PAK

**Keywords:** interventional radiology, trauma, pediatric

## Abstract

Objective

The aim of this study was to describe the outcome of the use of interventional radiological procedures (IRP) (angioembolization) in critically injured children.

Methods

A retrospective review of medical records of all children who underwent an IRP from January 2010 to December 2015 was done. Data were collected on a structured proforma and results are presented as mean with standard deviation and frequency with percentages.

Result

Eighteen patients were identified who underwent IRP during the study period. The mean age was 10.4 ± 4.3 years and 10 (55%) were males. Ten patients had a road traffic accident, four had a history of fall, one patient had glass cut pelvic injury, and two patients had blunt abdominal trauma, while one patient had bleeding secondary to hemipelvectomy. The genitourinary system was involved in five patients, liver in four, and spleen in two and pancreas in one patient. Bleeding was from branches of internal iliac artery in seven patients, hepatic artery in three patients, splenic artery in two patients, and middle colic artery in one patient, while one patient had blood oozing from the bone after hemi-pelvictomy. Four French vascular access sheath was placed under ultrasound guidance; this was followed by the placement of C1 catheter (Cordis, Miami, FL). After vessel identification, a 2.7F Progreat microcatheter (Terumo, Tokyo) was used for super-selective cannulation of the bleeding vessel. Intravascular coil, polyvinyl alcohol (PVA) particles, or gel foam was used for the embolization of bleeding vessels. No procedural complications were observed except minor oozing in one patient. One patient expired due to multiorgan dysfunction.

Conclusion

Angioembolization is a useful and relatively safe procedure in the management of vitally stable children with hemorrhagic abdominopelvic injuries. However, further studies may be needed to evaluate the efficacy and cost-effectiveness of this practice, especially in resource-constrained settings.

## Introduction

Childhood injury is one of the leading causes of death in the United States [1]. The incidence of childhood injuries and their associated morbidity and mortality are increasing in developing countries such as Pakistan [2]. Critically injured children often have injuries to multiple organs, sometimes with massive bleeding that requires immediate resuscitation and surgical or non-surgical interventions. Previously, operative management used to be the only option in such cases. But surgery in such patients has been found to be associated with high morbidity and mortality. In hemodynamically stable patients, less invasive procedures by interventional radiology (IR) come to the rescue and are not only lifesaving but also have lesser morbidity and the potential for full organ salvage [3-6]. IR has become a cornerstone of these minimally invasive procedures for critically injured adults and has the advantage of finding the source vessel causing hemorrhage and stopping hemorrhage by trans-arterial embolization under fluoroscopic guidance immediately [7]. There is less associated morbidity through these minimally invasive techniques. While the role of IR in critically ill children is increasing, there are still limitations and controversies as what is the best modality and when to use and when not to use [8]. A major limitation in Third World countries like Pakistan is the availability of skilled personnel and IR facility. There is no published data on the use of IR procedures for non-operative management of children with trauma, which can be lifesaving as well as reduce the morbidity. We hypothesize that IR is a successful intervention in trauma management in younger children without any major adverse effects. We share our experience of the use of IRPs in critically injured children admitted in a pediatric intensive care unit (PICU) and their outcomes.

## Materials and methods

A retrospective review of medical records of all children admitted with unintentional trauma (blunt/penetrating), who underwent an IRP from January 2010 to December 2015, was included in this study after obtaining approval from the ethical review committee (ERC) of the Aga Khan University Hospital. Any patient undergoing a conventional angiography to find the source of bleeding in a trauma patient was included in the study. Only those patients who were hemodynamically stable (on arrival or after resuscitation) and in whom angioembolization was performed were included in the study. All injuries were identified by computed tomography (CT) scan that was done after the stabilization of the patient. Injuries were graded using the Organ Injury Scaling Committee Guidelines on a scale of one to five according to CT findings. The dynamic intravenous contrast-enhanced abdominal CT included both arterial and venous phases. Uncontrolled bleeding was defined as continuous bleeding from any injured organ vessel, accompanied by tachycardia, persistently dropping hemoglobin of >2 g/dl over four to six hours and or hemodynamic instability.

The initial management of all trauma patients in our center is done according to the Advanced Trauma Life Support (ATLS) guidelines by a multidisciplinary trauma team. After the primary and secondary survey, initial stabilization is performed with intravenous fluids and or blood products followed by extended focused abdominal sonography for trauma (EFAST) and trauma series X-rays and CT scan when indicated. After stabilization and any intervention if needed, the critically injured patients are cared for in our multidisciplinary PICU.

Data were collected on a structured proforma and results are presented as mean with standard deviation and frequency with percentages.

## Results

In total, 18 patients underwent an IR procedure during the study period. The mean age was 10.4 ± 4.3 years and 10 (55%) were males. Trauma was the primary cause of injury in 17 (94%) children with the mean injury severity score of 15 (±5), while one patient had hemipelvectomy due to Ewing’s sarcoma (Table 1).

**Table 1:** Characteristics of 18 patients who underwent interventional radiology procedure IRP: interventional radiology procedure, IV: intravenous, ISS: injury severity score

Patient No	Age in years /Gender	Major organ involved	Grade of injury	ISS	Massive transfusion	Mean pre/post IRP hemoglobin	Length of stay	Complications	Alive/expired
1	9/M	Perineum	-	10	Yes	5/11.2	5	Bleeding from puncture site	Alive
2	4/F	Liver and stomach	IV	12		6/13.5	2	None	Alive
3	8/F	Kidney	-	15		3.8/8.2	1	None	Alive
4	11/M	Bladder and urethra	II	20		8/6.5	11	None	Alive
5	10/M	Splenic artery aneurysm	-	23		8.4/9.5	6	None	Alive
6	12/M	Spleen	IV	25		8.6/12	4	None	Alive
7	14/F	Liver	IV	20	Yes	5.8/13	4	None	Expired
8	12/M	Liver	III	9	Yes	7.9/12	2	None	Alive
9	13/M	Pancreas	IV	10		5.4/11	1	None	Alive
10	15/F	Kidney	IV	7		4.8/10	10	None	Alive
11	7/F	Pelvis	-	15		7.2/9	3	None	Alive
12	16/F	Pelvis	-	16	Yes	6.7/7.5	10	None	Alive
13	16/M	Pelvis	-	14		10/6.8	5	None	Alive
14	15/M	Pelvis	-	12		4.4/12.5	2	None	Alive
15	5/M	Pelvis	-	11	Yes	6.7/10.4	4	None	Alive
16	5/M	Pelvis	-	10	Yes	9/9.2	2	None	Alive
17	16/M	Liver	IV	23		3.7/8.8	10	None	Alive
18	16/M	Pelvis	-	22		4.8/7.8	8	None	Alive

**Figure 1:**
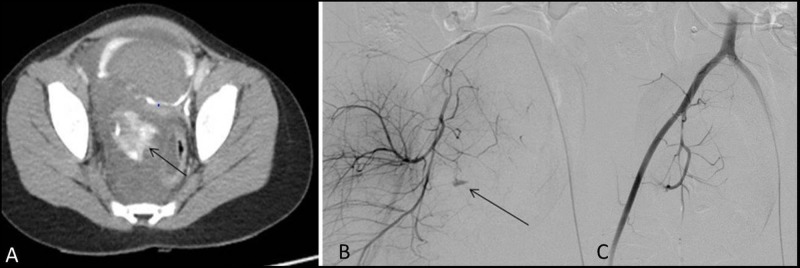
Child presenting with perineal injury due to glass A: Large pelvis hematoma with active extravasation (arrow) seen on CT. Bladder shows filling defect due to clot. B: Extravasation from branches of internal iliac artery (arrow). C: Successful embolization of abnormal vessel with no further extravasation CT: computed tomography

Ten patients had met with road traffic accident (RTA), four patients had a history of fall, one patient had abdominal trauma due to hitting of bicycle handle, other had swing rod injury on the abdomen, and one patient had bleeding secondary to hemipelvectomy, while one patient had glass cut injury to the pelvis (Figure 1).

In this type of injury, 10 patients had a blunt injury, five had runover injury (crushing), two had a penetrating injury, and one was a post-operative hemi-pelvictomy patient. The genitourinary system was involved in five patients, liver in four, and spleen in two, and pancreas in one patient. Spleen and pancreas had grade IV injury, while liver had grade V in two and grade III in one patient. Three patients had a mild traumatic brain injury (TBI) and Glasgow coma scale (GCS) of 12-13 on presentation. Two patients had moderate TBI and GCS of 8-12, while rest of them had normal GCS on arrival. All patients underwent computed tomographic angiography (CTA) to assess the severity of the injury and find the focus of bleeding. CT scan was only done after stabilizing the patient hemodynamically. The mean hemoglobin level before and after the intervention was 6.45 ± 1.9 g/dl and 9.9 ± 2.1 g/dl. The mean time from presentation to intervention was 10.7 ± 10.7 hours (Table 2).

**Table 2:** Time interval between presentation and intervention and its relationship with length of stay

Serial number	Time between presentation and IRP (hours)	Length of stay (days)
1	5	5
2	11	2
3	8	1
4	5	11
5	48	6
6	3	4
7	11	4
8	5	2
9	6	1
10	10	10
11	18	3
12	12	10
13	4	5
14	5	2
15	6	4
16	4	2
17	8	10
18	24	8

Indication for involving interventional radiologist was uncontrolled bleeding (hemodynamic instability and a persistent drop in hemoglobin in the presence of proven or suspected vessel injury) not responding to transfusion. Bleeding was from branches of internal iliac artery in seven patients, from the hepatic artery in five patients, the splenic artery in two patients, and middle colic artery in one patient, while one patient had blood oozing from the bone after hemi-pelvictomy (Table 3).

**Table 3:** Cause of trauma and type of arterial injury

Patient number	Age/Gender	Cause of trauma	Arterial injury
1	9/M	RTA	Internal iliac
2	4/F	RTA	Hepatic artery
3	8/F	Fall from roof	Hepatic artery
4	11/M	RTA	Internal iliac
5	10/M	RTA	Splenic artery
6	12/M	RTA	Splenic artery
7	14/F	Fall from roof	Hepatic artery
8	12/M	RTA	Hepatic artery
9	13/M	Bicycle handle injury	Middle colic artery
10	15/F	Glass cut injury	Internal iliac
11	7/F	RTA	Internal iliac
12	16/F	RTA	Renal artery
13	16/M	RTA	Internal iliac
14	15/M	Blunt trauma by swing rod	Splenic artery
15	5/M	RTA	Internal iliac
16	5/M	RTA	Internal iliac
17	16/M	RTA	Hepatic artery
18	16/M	Hemipelvectomy	Internal pedundal Artery
RTA = Road traffic accident			

Procedures were performed under general anesthesia in all patients. A 4Fr vascular access sheath was placed under ultrasound guidance, and this was followed by the placement of C1 catheter (Cordis, Miami, FL). After the vessel was identified, a 2.7F Progreat microcatheter (Terumo, Tokyo) was used for super-selective canulation of the bleeding vessel. Intravascular coil, polyvinyl alcohol (PVA) particles, or gel foam was used for the embolization of bleeding vessel. The embolization material used included a platinum coil with PVA particles in 10 patients, platinum coils alone in four patients, PVA particles alone in three patients, and gel foam with PVA particles in one patient. The success rate to control bleeding was 100%. No immediate post-intervention complications were observed except one patient who had oozing from the vascular access site. The average length of stay in pediatric intensive care unit (PICU) was 4.2 days (range: 1 to 11 days). One patient expired during the PICU stay due to multiorgan dysfunction. The time of intervention was between 8 am to 5 pm in seven patients, between 5 pm and 12 am in three, and between 12 am to 8 am in four patients.

## Discussion

The non-operative management of trauma, already the standard of care in adults, continues to gain acceptance in children [3,9]. It is often sufficient in children for hepatic, splenic, and renal injuries and adjunctive procedures as angioembolization are less often required. The challenges in interventional procedures are related to the smaller size of pediatric arteries and hence the chances of complications increase. We have encouraging results, including short duration from presentation to intervention, success in IRP, and reduced length of stay, which emphasize the use of IRP in critically injured children affected by severe polytrauma. The mean age in our cohort is 10 years, which shows that IR still has limitations in younger children because of the small vascular size and meticulous care and expertise needed for these patients. The youngest child in our series was four years old and two children were five years old. In a similar study by Lin et al. who studied the use of IR in blunt pediatric renal trauma, the mean age was 10 years. The mechanism of injury (RTA and domestic trauma) was also similar to their series and others [10-11]. Another series from South Africa has shown successful results in very young children with renal trauma with the help of non-operative management [12]. Similar success with IR has been shown in blunt hepatic and splenic trauma in adults, while in our series, four patients had a liver injury and two had splenic trauma [9,13-14]. Perineal/pelvis and urologic tract were injured in 11 of our patients. The bleeding from these areas is very difficult to control which again emphasizes the need and importance of IR in these injuries [15]. The mean hemoglobin levels before intervention were low and six patients received massive transfusion in first 24 hours but after the procedure; this stabilized with mean hemoglobin of 9.9 ±2.1 g/dl. None of our patients developed any complication after the IR procedures except one with minor oozing of blood from the insertion site. The mean length of PICU stay in our cohort was four days which again emphasizes that timely IR intervention can be very helpful in salvaging these patients and decreasing the morbidity as well as cost. This could also be the reason that all these patients were resuscitated adequately before IRP and were hemodynamically stable and so there is a possibility of selection bias as patients who were hemodynamically unstable were not selected for IRP. Currently, there are no criteria as to which patient should undergo operative versus non-operative management in children and there are certain controversies in pancreatic trauma. But we hope that as our experience of the use of IR increases in this population, its use will spread. As the trauma and its associated mortality and morbidity increases in our part of the world, this modality can be lifesaving.

This is the first report from our country describing the use of non-operative measures like IRPs for children with critical arterial injuries with great success; however, our study has the limitation of being a single-center, retrospective report with a limited sample size. Four interventional radiologists performed the procedures and they had variable experience ranging from five to 20 years; hence, theoretically, outcomes may depend on their experience; although in our series, most of them very good.

Nevertheless, it serves as an initial report with encouraging and successful results of IRP in critically injured children.

## Conclusions

Angioembolization is a useful and relatively safe procedure in the management of vitally stable children with hemorrhagic abdominopelvic injuries. However, further studies may be needed to evaluate the efficacy and cost-effectiveness of this practice, especially in resource-constrained settings.
